# Acne vulgaris: A review of the pathophysiology, treatment, and recent nanotechnology based advances

**DOI:** 10.1016/j.bbrep.2023.101578

**Published:** 2023-11-23

**Authors:** Mallikarjun Vasam, Satyanarayana Korutla, Raghvendra Ashok Bohara

**Affiliations:** a Chaitanya (Deemed to Be University)-Pharmacy, Hanamkonda, Warangal, Telangana, India; b Centre for Interdisciplinary Research, D.Y. Patil Educational Society, Kolhapur, India; c CÚRAM, SFI Research Centre for Medical Devices, University of Galway, Ireland^1^

**Keywords:** Acne vulgaris, Acne treatment, Acne inflammation, Acne pathogenesis, Topical retinoids, Systemic, Oral antibiotics

## Abstract

**Background:**

Globally, Acne Vulgaris is a widespread, chronic inflammatory condition of the pilosebaceous follicles. Acne is not fatal, but depending on its severity, it can leave the sufferer with scars, irritation, and significant psychological effects (including depression). In the current review, we have included various factors for acne and their treatment explained. It also narrated the current medicament and the new investigation dosage forms with clinical phases information provided.

**Main body of the abstract:**

Acne's pathophysiology involves four important factors: excessive sebum production, hyperkeratinization of pilosebaceous follicles, hyperproliferation of propionibacterium acnes (P. acnes), and inflammation. Identifying both inflammatory (Papule, pustule, nodule, and cyst) and non-inflammatory (black heads, white heads) acne lesions is necessary for diagnosing and treating acne vulgaris.

**Short conclusion:**

In this review, traditional therapy approaches such as topical (i.e., retinoids and antibiotics), systemic (i.e., retinoids, antibiotics, and hormonal), and physical therapies are briefly discussed. In addition, we highlight the issues posed by P. acne's resistance to the antibiotics used in commercially available medications and the necessity for novel therapeutic techniques. Finally, we examined a few innovative acne therapies pending clinical trial approval and commercial acne medications.

## Highlights

• Acne Pathophysiology Insights.
• Nanotech Advances in Acne.
• Antibiotic Resistance Addressed.
• Comprehensive Treatment Review.
• Dermatology's Future Explored.

## Graphical abstract

**Figure:**
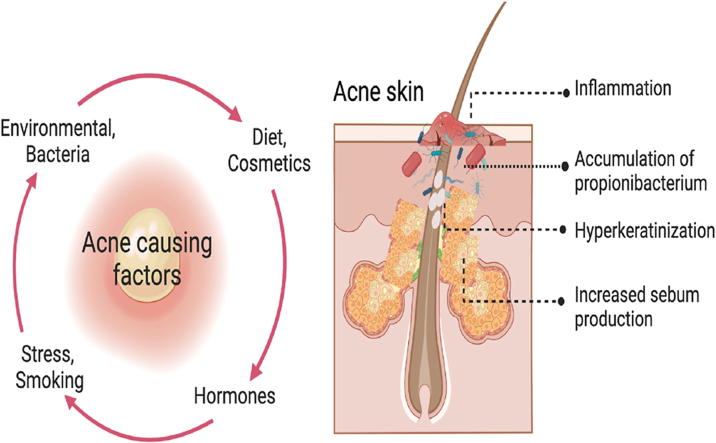

## 1 Background

Acne (also known as Acne Vulgaris) is a persistent chronic skin inflammatory condition of the pilosebaceous follicles that affects people all over the world [[1], [2], [3]]. Acne is projected to affect 9.4 % of the global population, ranking it eighth among skin diseases. Acne affects more than 85 % of teenagers, and the disease can persist into adulthood which often occurs in females and accounts for two-thirds of dermatologist consultations for acne [4]. The unique lesions can be characterised as either non-inflammatory (open/black and closed/white comedones) or inflammatory (papules, pustules, nodules, and cysts), leading to scar development and pigmentation on the skin, necessitating prolonged and persistent therapy [5]. Typically, lesions are observed on the face, neck, upper back, and chest. There are several forms of acne, such as neonatal and infantile acne, occupational acne, acne conglobata, acne fulminans, acne mechanica, excoriated acne, chloracne, and acne induced by drugs (caused, for example, by anabolic steroids, corticosteroids, isoniazid, lithium, and phenytoin). These variations resemble acne vulgaris clinically and histologically, but the clinical situation, severity can distinguish them, and accompanying symptoms [6]. Acne can't be stopped from occurring or cured, but it can be treated quite successfully. Moreover, acne is also connected with significant financial expenses.

It is noteworthy that the number of research papers and scientific findings relevant to acne treatment regimens continues to increase, given the enormous interest in various research breakthroughs in this area. Much study has been conducted on acne, both in terms of the disease process and the numerous therapy approaches. The most recent advances in acne therapy involve the use of combination treatments that address the numerous pathogenic factors that are at the root of the condition. The most recent literature on acne treatment, which described several topical applications, novel treatments, and emerging treatments for successful acne-targeted therapy, includes topical retinoids that normalize abnormal hyperkeratinization in the infundibulum and novel topical retinoids with anti-inflammatory properties [[7], [8], [9], [10]]. The present article aims to give a clear picture of acne types, their pathogenesis, and biological pathways of various acne types and also discusses conventional treatment medication strategies in detail. The novelty of the current review highlighted the information from recent research and review along with in detailed description of the treatment combination. We also highlighted the dosage forms which are in various trial phases, along with successful and commercially available dosage forms in the market. Besides, we will review the recent developments and discuss some potential clinical trial studies in detail.

## 2 Main text

### 2.1 Factors contributing to acne pathogenies and types of acne

**Fig. 1:**
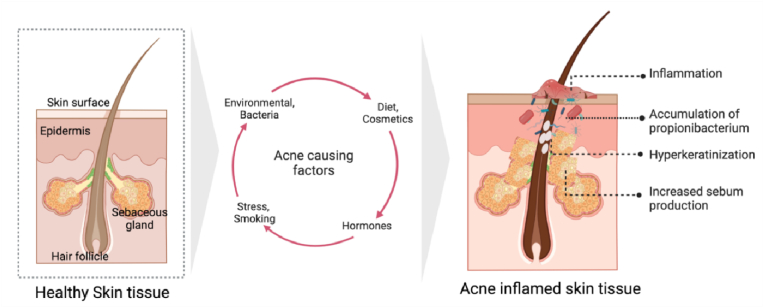
Schematic illustration of healthy/normal skin tissue vs acne inflamed skin tissue, various factors (environmental, bacterial, diet, stress, smoking and relevant hormonal imbalance among others) contributing to the formation and development of acne.

The following elements are thought to be relevant in the classical aetiology of acne vulgaris (see Fig. 1). Genetics, environmental variables (temperature, pollution, humidity, sun exposure, mineral oils/halogenated hydrocarbons), nutrition, hormonal state, stress, smoking, comedogenic medicines such as androgens, halogens, corticosteroids, bacteria, and cosmetics [7] may cause, worsen, or exacerbate acne [8,11]. Acne Vulgaris (AV) typically causes discomfort, emotional suffering, deformity, and possibly permanent scars. In addition to this, patients may have feelings of anxiety and embarrassment, both of which contribute to a mentally depressed state.

Due to the aforementioned exposome factors, the pathogenesis of acne is multifactorial, which involves the excess production of sebum, hyper proliferation of the bacteria colonization known as cutibacterium acnes (previously known as propionibacterium acnes), abnormal hyperkeratinization of the pilosebaceous follicles, and inflammatory mechanisms are the four primary causes for acne [12]. The next section will describe the various causative factors and pathogenesis (see Fig. 1).

### 2.2 Causative factors and pathogenesis of acne vulgaris

Several causal factors are believed to play a key part in the traditional aetiology of acne vulgaris. As discussed previously, chronic acne skin diseases are caused by increased sebum excretion rates, endocrinological factors such as androgens, aberrant keratinization of the follicular infundibulum, bacterial infection proliferation, and consequent inflammation (see Fig. 1).

a) ***Increase in Sebum production***: An increase in sebum production in the hair follicles is one of the most significant causes of acne formation. According to Gollnick et al. androgen hormones, specifically testosterone and Insulin Growth hormone (IGH-1), increase sebum synthesis and secretion [13]. There is a clear correlation between increased sebum production and the severity and frequency of acne lesions; as a result, it is a significant element that should be considered in patients suffering from acne vulgaris [[14], [15], [16]].
b) ***Hyperkeratinization abnormalities of the pilosebaceous follicles***: Generally, the healthy follicles often shed single-cell keratinocytes into the lumen, which are then ultimately eliminated. However, in acne patients, keratinocytes hyper proliferate and are not shed into the lumen, which leads to the accumulation of irregular desquamated corneocytes in the pilosebaceous follicles coupled with lipids and monofilaments [[17], [18], [19]].
c) ***Hyper proliferation of propionibacterium acnes (P. acnes)***: Propionibacterium, which plays a substantial part in the pathophysiology of inflammatory acne, is an additional acne-causing agent. Cutibacterium acnes, formerly known as propionibacterium acnes, is an anaerobic, lipophilic, gram-positive pathogen that prefers to colonise in sebaceous follicles because they produce large amounts of sebum and provide excellent anaerobic habitat for bacterial growth [20]. P. acnes secretes a lipase enzyme that metabolizes the triglycerides of sebum into glycerol and fatty acids, which can lead to the formation of comedones and inflammation on the skin [21].
d) ***Inflammation acne***: On continuation to the above P. acne process, when the immune system detects P. acnes, the inflammatory process begins. P. acnes has a strong inflammatory effect, which may produce chemostatic agents like lymphocytes, neutrophils, and macrophages. In addition, these conditions cause follicular damage, rupture, and the release of germs, fatty acids, and lipids into the dermis layer. These mechanistic processes will produce inflammatory lesions such as ulcers (pustules, nodules, cysts and papules). Non-inflammatory lesions are smaller and less pus-filled than inflammatory lesions [22,23]. In addition, it was discovered that neutrophils produce reactive oxygen species (ROS), which damage the follicular epithelium and contribute to acne inflammation. This causes the follicular substances to be expelled into the dermis, resulting in a variety of inflammatory acne lesions [24].
e) ***DNA Methylation***: Under environmental stress, epigenetic modification, which represents the intersection of genetics and the environment, can alter the expression of genes. DNA methylation, one of the well-studied forms of epigenetic modification, is gaining increasing attention in the field of dermatology for its function in the mechanisms of inflammatory, autoimmune, and cancerous skin diseases. DNA methylation has been shown to play a role in the pathogenesis and progression of inflammatory skin diseases such as hidradenitis suppurativa, atopic dermatitis, psoriasis, and other inflammatory skin disorders. Epigenetics plays a significant role in the development of acne vulgaris and may offer insights into its molecular mechanisms and potential therapeutic approaches [25].

### 2.3 Types of acne lesions

**Fig. 2:**
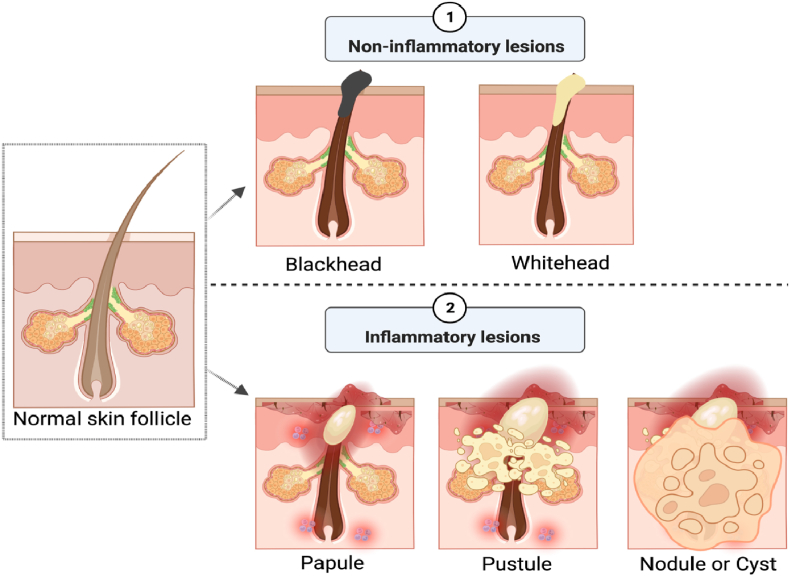
Schematic illustration of major distinguishing of the two types of lesions (non-inflammatory, inflammatory) and their pathogenies.

Acne is classified into several forms, including acne conglobate, acne rosacea, acne fulminans, acne cosmetica, acne excoriee (picker's acne), acne medicamentosa, acne chloracne, and acne mechanica [26,27]. Nonetheless, acne vulgaris is the most prevalent form of acne, accounting for 99% of all acne cases. It is differentiated by two types of lesions: non-inflammatory, open and closed comedones, as well as inflammatory papules, pustules, nodules, and cysts (see Fig. 2). The comedones are of two types: a comedo that is closed is a whitehead, while another that is open is a blackhead type [28].

**Blackheads**: Blackheads are non-inflammatory acne lesions that develop on the skin due to excess oil and dead skin cells obstructing hair shafts. A blackhead is referred to as an open comedo because the skin surface remains exposed and has a dark look, such as black or brown. Blackheads are mild acne that usually appears on the face, arms, chest, neck, back and shoulders.

**Whiteheads:** Whiteheads are small bumps and non-inflammatory acne lesion that develops on the skin when oil, bacteria and skin cells block the opening of hair follicle pores. Whiteheads are referred to as closed comedones since the bumps are closed and white. Whiteheads can develop anywhere on the body, but they are most frequent in the T-zone, which includes the nose, chin, and forehead.

**Papules:** Inflammation is the response of healthy skin tissue to bacteria, excess oil production, and excess androgen activity, and its symptoms include swelling, heat, redness, and pain. These inflamed lesions are known as papules and are considered an intermediary step between non-inflammatory and inflammatory lesions (see Fig. 2). Papules show on the skin as a little pink lump typically less than 5 mm in diameter and not pus-filled.

**Pustules:** Pustules are small bumps and an inflammatory lesion that occurs on the skin by clogging the pores with excess oil and dead skin cells. Pustules are inflammatory lesions that contain fluid or pus in their centre. Often, they manifest as white pimples surrounded by red, irritated skin. Pustules can form on any part of the body, although they are most prevalent on the shoulders, chest, back, face, neck, underarms, pubic region, and hairline.

**Nodules:** Acne nodules are a severe form of inflammatory acne that develops when the pores become clogged by bacteria, excess oil and dead skin cells. This type of combination usually causes whitehead or blackhead comedones, but if the infection penetrates underneath the surface of the skin and affects the pores as well as the surrounding area to become red and swollen and appear as a small bump. Acne nodules are not treatable with over-the-counter medications alone and might remain for weeks or months. Nodular acne is similar to papule acne, but its diameter is bigger than 5–10 mm, and it often develops on the face's jawline or chin.

**Cysts:** Cystic acne is a severe kind of inflammatory acne that appears beneath the skin due to blocked pores caused by the accumulation of bacteria, dry skin cells, and oil (see Fig. 2). People with the oily skin of all ages are most affected. Cyst typically appears as large white/red painful lesions filled with pus, sometimes leading to scars. Cystic acne can appear anywhere on the body, although it most frequently affects the face, arms, shoulders, back, chest, and neck. Most people with cystic acne experience both inflammatory and non-inflammatory acne symptoms.

#### 2.3.1 Prevalence of acne

According to the Global Burden of Disease Study, acne vulgaris is the eighth most common skin disease worldwide, with an estimated global prevalence of 9.38 % for all age groups. The prevalence of acne varies among different countries and age groups, with estimates ranging from 35 % to close to 100 % of adolescents experiencing acne at some point [29]. According to Shah et al. the prevalence of adult acne was found to be 0.74 % in their study on the clinico-epidemiological pattern of acne vulgaris in adult patients. The high prevalence of acne in adults may be attributed to increase reporting to healthcare facilities and improved awareness. High glycemic diets may contribute to the high prevalence of acne [30].

## 3 Conventional medications for acne treatment

Amongst the available treatment options for acne vulgaris primary objective is to manage and treat existing lesions by controlling the sebum secretion, abnormal hyperkeratinization of the pilosebaceous follicles and propionibacterium infection. As a result, the main treatment options for acne include anti-inflammatory drugs and antibacterial drugs [14,[31], [32], [33]] administered either through topical or systemic or oral route of administration and by physical way by using non-drug treatments such as optical therapy, cryotherapy, comedone extraction, cyroslush therapy and intralesional corticosteroids. On the other hand, combinational treatment (topical and oral route) is more effective in acne pathogenesis [14,31].

### 3.1 Topical treatments

Topical therapies have the advantage of being given topically to the diseased area, hence minimising systemic absorption and increasing pilosebaceous follicular unit exposure. Numerous formulations of topical preparations include creams, gels, lotions, solutions, and washes. Mild to moderate acne is typically treated with topical medicines [34,35]. Besides retinoids, antibiotics are used topically to treat patients suffering from acne. Skin irritation is a common side effect of topically administered anti-acne medications. Topical treatment may last 6–8 weeks or continue for many years [36].

#### 3.1.1 Retinoids

Topical retinoid therapy is the most used first-line treatment for non-inflammatory and inflammatory acne [14,37]. The primary objective of retinoids is to reduce sebum production, regulate the growth of comedones, rebuild the damaged epithelium layer, treat hyperpigmentation and scarring, reduce the creation of acne lesions, and control the growth of existing comedones. Nevertheless, this therapy is a lengthy procedure that requires more than three months to cure acne. The disadvantages of topical retinoids include dryness and skin irritation. Tretinoin, Adapalene, and Tazarotene are often used as retinoids for acne therapy.

a) ***Tretinoin:*** It is a vitamin A derivative form of a drug and possesses anti-inflammatory properties. This medication is usually used in conjugation with other retinoids in the acne vulgaris treatment to normalize the epithelial layer, hence preventing the occlusion of pilosebaceous units and reducing sebum production. It has been used as a topical therapy for acne for over three decades. It is available in the market as creams, gels, and ointment forms for acne treatment [38]. When applied topically, the side effects are mild and cause sun sensitivity and redness but cause headache, skin dryness, hair loss, itching and muscle pains when consumed orally in Leukemia treatment [39].
b) ***Adapalene*:** Primarily used topical retinoid drug for treating mild-moderate acne patients. It has more advantages than other retinoids like Tretinoin and Tazarotene and is considered a first-line therapy for acne treatment [40]. It helps to reduce the hyperkeratinization of pilosebaceous follicles, inflammation caused by acne. The side effects are minimal such as redness, irritation, and itching on the skin [41].
c) ***Tazarotene*:** It is one of the novel topical retinoids used for acne vulgaris treatment. After tretinoin or adapalene treatments have failed to produce results, tazarotene is considered a second-line treatment. It helps to reduce hyperkeratinization and hyperproliferation of Propionibacterium Acnes in the epithelial layer of acne patients [42,43]. Tazarotene is combined with benzoyl peroxide or antibiotics for treating inflammatory acne as it also has inflammatory properties; combinational therapy is more effective than monotherapy, yet skin irritation and redness are the adverse effects of Tazarotene [44].

#### 3.1.2 Antibiotics

Topical antibiotics are generally used to treat mild to moderate inflammatory acne. They have anti-P. acnes activity and hence act on the skin's surface to diminish inflammatory lesions [45]. Due to their ineffectiveness and undesirable side effects, not all topical antibiotics (chloramphenicol and tetracycline) are used to treat acne. Erythromycin and clindamycin are the two most commonly used topical antibiotics for treating acne. Using topical antibiotics with benzoyl peroxide and topical retinoids to prevent bacterial resistance is a more effective strategy than monotherapy [46]. When applied directly on the skin, topical antibiotics reduce P. acnes colonization and inflammation in acne patients [47]. Here are the few topically used antibiotics in acne treatment are.

a) ***Erythromycin*:** It is a topical antibiotic used in treating acne patients. Erythromycin, when administered topically on the skin, helps to reduce the colonization of P. acnes in pilosebaceous follicles and reduce inflammation. Researchers observed that erythromycin has 60 % bacterial resistance, which is less desirable and leads to the creation of new topical antibiotics in the future.
b) ***Clindamycin*:** It is a semi-synthetic topical antibiotic used for treating acne patients. Clindamycin also has properties similar to erythromycin, inhibiting the P. acnes on the surface of the skin and reducing inflammation. Topical antibiotics monotherapy should be avoided for treating acne vulgaris and encourage combinational treatment for better use.

#### 3.1.3 Combinational topical treatments

Combinational topical therapy has many benefits compared to monotherapy treatment. Other topical treatments used during combinational therapy for acne are benzoyl peroxide, salicylic acid, niacinamide, azelaic acid and dapsone.

a) ***Benzoyl Peroxide (BPO):*** Benzoyl Peroxide acts as a topical non-antibiotic disinfectant and also exhibits antibacterial properties, which are used for treating mild to moderate acne [48]. BPO produces free oxygen, which helps in the degradation of bacterial proteins, hence proving the bactericidal action against P. acnes [47,49]. Initially, 6–8 weeks acne patients can be treated with BPO monotherapy, but for effective results, BPO is combined with topical antibiotics to minimize P. acne species resistance and promotes therapeutic efficacy [50]. The most common BPO adverse effects are itching, peeling, irritation and redness on the skin may occur [51,52].
b) ***Salicylic acid:*** Salicylic acid is a beta hydroxyl compound that has anti-inflammatory, fungistatic and bacteriostatic properties. It reduces acne by exfoliating the skin and keeping pores clear when applied topically. However, salicylic acid is safe to use but sometimes causes skin irritation, itching and dryness [53]. It is mainly used to treat mild acne. Numerous over-the-counter drugs are available for acne treatments, which majorly contain salicylic acid [54]. Supramolecular Salicylic Acid treatment significantly improved the acne patients and modulates the microbial diversity of patients toward like individuals with no facial acne [55].
c) ***Niacinamide:*** Niacinamide is a form of vitamin B3 called nicotinamide (composed of niacin/nicotinic acid). It treats acne patients to lessen the sebum/oil secretion and shield the skin from developing acne [56]. Niacinamide has anti-inflammatory properties and is used to treat mild to moderate acne. It helps acne patients recover from sun damage, fine lines, redness, and wrinkles. Due to its beneficial/therapeutic properties, niacinamide is used in acne medications and other skincare products [57,58].
d) ***Azelaic acid*:** Azelaic acid is a naturally occurring substance derived from barley and wheat with antibacterial, anti-inflammatory, anti-keratolytic, and anti-oxidative activities [59]. Combination therapy for acne is more effective than azelaic acid monotherapy. Azelaic acid's most common side effects are skin redness, burning, difficulty breathing, and itching. Azelaic acid is used to cure acne and treat other skin disorders, such as skin whitening and hyperpigmentation [60].
e) ***Dapsone:*** Dapsone (diaminodiphenyl sulfone) has antibacterial and anti-inflammatory properties, but its exact mode of action in treating acne is still unknown. Recently, it has been proposed that dapsone's antimicrobial, immunomodulatory and anti-inflammatory properties may be responsible for treating mild to moderate acne [61]. Both inflammatory and non-inflammatory acne lesions can be reduced with dapsone gel (5 %). Because of its cheaper price, this agent is more suitable for usage in developing nations. Nevertheless, it is not advised as a first-line treatment for treating acne vulgaris [62,63].

### 3.2 Systemic treatment

As the microcomedo plays a crucial role in the growth of both inflammatory and non-inflammatory lesions, topical retinoids should be considered as first-line therapy for treating acne. Oral systemic treatment is preferred when acne patients do not react to topical treatment or if it appears as nodular lesions on the skin or with scars. Systemic treatment is essential for acne patients to prevent social humiliation and psychological embarrassment. Oral antibiotics, hormonal medicines, and isotretinoin are the most used systemic treatments for treating acne vulgaris [64].

#### 3.2.1 Retinoids

Isotretinoin is a type of retinoid mostly preferred in systemic treatment, and it is also a vitamin A derivative. Formerly it was the only medicine that could suppress acne over the long term and is used as a first-line treatment for severe nodular or inflammatory acne. Patients with mild to moderate acne who have previously failed to respond to oral or topical medications may also find it helpful. It is also regarded as a first-line treatment for severe acne on the face and trunk, acne that leaves scars, and acne that leads to being psychologically ill [65].

Isotretinoin is the current only medicine available in the market that shows an effect on all four pathogenic causes of acne [52,66]. It causes sebaceous gland de-differentiation, which results in decreased sebum production and a change in the cutaneous bacterial flora environment, ultimately reducing the colonization of P. acnes in the hair follicles, Keratinocytes are also shed as a result of this [67,68]. The duration of isotretinoin therapy is typically administered over 16–24-week period. Because of the adverse effects of isotretinoin, patients must be continuously monitored [69].

#### 3.2.2 Antibiotics

Oral antibiotics are usually prescribed for acne that is moderate to severe, inflammatory, resistant to previous topical treatments, or covers a large body area [70,71]. Oral antibiotics like erythromycin, clindamycin, azithromycin, roxithromycin, fluoroquinolones (levofloxacin), tetracyclines (doxycycline, minocycline, and lymecycline), and co-trimoxazole are often used to treat acne [14,52].

These antimicrobial drugs suppress P. acnes growth and the development of inflammatory mediators produced by P. acnes. The capacity of the antibiotic to reach the lipid environment of the pilosebaceous follicles in the dermis, where P. acnes colonizes, determines the efficacy of the treatment [72]. Tetracycline's are widely used for treating acne because they are effective, contain anti-inflammatory and antibacterial properties and are economical. Doxycycline and minocycline are chosen over tetracycline's because they have anti-inflammatory properties, cause less GI discomfort and are more lipid soluble, allowing them to penetrate the pilosebaceous follicle more effectively [73]. There is no sufficient data in the literature to prove that minocycline and doxycycline are more efficient than tetracycline's. Furthermore, tetracyclines have been associated with reduced resistance in P. acnes than macrolides [74].

The researchers haven't conducted numerous studies to determine the efficacy of azithromycin for treating acne. P. acnes levels are reduced due to the minimal anti-inflammatory effects of erythromycin and clindamycin [69]. In acne vulgaris treatment, frequently administering these antibiotics for a long duration leads to increased resistance and limited use of these agents. Nowadays, combinational treatment is chosen for a better efficacy rate and to minimize resistance. Oral antibiotics are conjugated with topical agents like benzoyl peroxide or retinoids in treating acne, and the duration of treatment is limited to 12 weeks [14,75].

#### 3.2.3 Hormonal treatment

In adult females and adolescents, hormonal treatment was chosen as an alternative method for treating acne. Hormone therapy can be used to treat the effect of androgen on sebaceous glands because sebaceous glands are androgen-dependent [76]. Often, these hormones are delivered as oral contraceptive tablets. These contraceptive hormone pills inhibit the sebum production that is first stimulated by testosterone. It increases the production of sex hormone-binding globulin, which decreases the amount of physiologically active free testosterone in women's bodies [77].

Acne in women can be treated with oral contraceptive pills alone or in conjugation with other treatments. Since the beneficial effects of hormonal medicines are apparent only after a 3–6 month treatment period, the most recommended treatment period for acne in females with hormonal anti-androgens is at least 12 months, if not more [78]. Spironolactone is an androgen receptor blocker combined with oral contraceptives to reduce inflammation caused by acne in females [14].

## 4 Recent advancements and commercial products

Recent scientific advances in the knowledge of acne's complexity have revealed new possibilities for study and development, such as inhibiting the mechanistic pathway or process involved in acne formation. Receptors, cytokines, chemokines, and other proinflammatory mediators were targeted in recent studies to control these pathways. In addition, therapeutic components of nutrition, the skin microbiome, the patient's genetics, and follicle-dwelling bacteria play a crucial part in acne therapy [79]. The use of medicines that release nitric oxide (NO) is one of the novel strategies that have the potential to be fruitful in acne management. NO has many functions, including highly effective anti-inflammatory, antibacterial, and antioxidant actions [80]. Isotretinoin (ISO) is a potent derivative of vitamin A used to treat acne. It has the potential to achieve long-lasting remission of acne. However, it is known to have side effects such as teratogenicity, skin reactions, ocular reactions, changes in blood indicators, and occasional Acne Fulminans. It is important to focus on minimising these side effects by considering combination medication and adjusting the dosage. This will help improve the treatment experience for patients [81]. Despite the significant increase in understanding of the causes of acne that have occurred over the past 20 years, many efforts have been made to develop novel therapy regimens specifically aimed at the elimination of acne. Some of the ongoing or latest clinical trials data has shown in Table 1 (the data obtained from ClinicalTrials.gov).

**Table 1:** Recent or ongoing clinical trial investigations for the treatment of Acne vulgaris.

NCT number	Interventions	Clinical trial phase/Stage	Outcome Measures	Sponsor/Collaborators
NCT02656485	Drug: B244 & Placebo	Phase 1 Phase 2	• Safety (Treatment related adverse events in patients) • Efficacy	• AOBiome LLC
NCT01694433	Drug: Calcipotriene & Placebo	Phase 2 Phase 3	• Lesion counts (Total, inflammatory and non-inflammatory) • Acne severity as assessed with the investigator's global assessment (IGA)	• University of California, Los Angeles • National Institute of Arthritis and Musculoskeletal and Skin Diseases (NIAMS)
NCT01936324	Drug: Olumacostat Glasaretil Gel, 7.5 % Other: Olumacostat Glasaretil Gel, Vehicle	Phase 1 Phase 2	• Mean absolute change in acne lesion counts (inflammatory and non-inflammatory)	• Dermira, Inc. • Eli Lilly and Company
NCT03761784	Drug: S6G5T-3 Drug: S6G5T-8	Phase 3	• % participants achieving an IGA score of clear or almost clear • Change from baseline in inflammatory, non-inflammatory lesion counts	• Sol-Gel Technologies, Ltd
NCT01910064	Drug: GK530G	Phase 3	• Local Tolerability (Erythema) • Local Tolerability (Scaling) • Local Tolerability (Dryness) • Local Tolerability (Pruritus) • Local Tolerability (Stinging/Burning) • % changes from baseline in total lesion counts	• Galderma R&D
NCT02566369	Drug: CD5789 (trifarotene) 50 μg/g cream Drug: Placebo cream	Phase 3	• Investigator global assessment (IGA) success rate at week 12	• Galderma R&D
NCT04594759	Drug: Isotretinoin	Phase 1 Phase 2	• Number of participants that showed improvement in their visible acne	• Medical University of South Carolina
NCT01628549	Drug: 50 mg P005672–HCl Drug: Placebo Drug: 100 mg P005672–HCl	Phase 2	• The absolute change from baseline in the inflammatory, non-inflammatory lesion count	• Almirall, S.A. • Allergan
NCT02720627	Drug: cortexolone 17#-propionate	Phase 2	• Change in HPA axis response as measured by CST • Evaluate trough plasma concentrations	• Cassiopea SpA
NCT04943159	Drug: Afamelanotide	Phase 2	• The change in number of facial inflammatory acne- related lesions	• Clinuvel Pharmaceuticals Limited
NCT02815280	Drug: FMX-101, 4 % minocycline foam Drug: Vehicle Foam	Phase 3	• Absolute change from baseline in the inflammatory lesion count	• Vyne Therapeutics Inc.
NCT02998671	Biological: CJM112 Other: Placebo	Phase 2	• Total Inflammatory Facial Lesion Count • Pharmacokinetics (PK): Serum Trough Concentrations of CJM112	• Novartis

Researchers have conducted many studies on acne, but only a handful of acne treatments have passed clinical testing and are now available to consumers. The acne therapies currently available on the market can be applied topically or systemically, but very few combine the two methods. The following is a list of a few commercial products (see Table 2) available worldwide.

**Table 2:** List of recent commercial products available for acne treatment.

Brand name	Drug class	Acne type	Route of administration	Marketed by
Benzamycin	erythromycin and benzoyl peroxide- Macrolide/antiseptic combination	Mild to moderate acne	Topical-Gel form	Dermik Laboratories
Differin	Adapalene-Retinoids	Mild to moderate acne	Topical-Gel form	Nestle pharmaceuticals
Solodyn	Minocycline-tetracycline antibiotics	acne	oral	Teva pharmaceuticals
Absorica	Isotretinoin-retinoids	Severe acne	oral	Hoffmann-Roche pharmaceuticals
Winlevi	Androgen Receptor inhibitors	acne	Topical cream	Cassiopea pharmaceuticals
Vibramycin	Tetracyclines, antimalarials-	acne	oral	Pfizer pharmaceuticals
Accutane	Isotretinoin-systemic	Severe acne	oral	Hoffmann-La Roche pharmaceuticals

## 5 Conclusions

Acne is a prevalent inflammatory skin condition that frequently causes depression and social embarrassment, mostly in adults. The aforementioned four key pathogenic factors are the causative aspects in the development of acne; this pathological process provides researchers clues to develop various therapeutics for acne management. In order to maximise the effectiveness of both existing and new treatments, it is essential to have a thorough understanding of the irritants that cause the formation of microcomedones and the transformation of non-inflammatory lesions into inflammatory lesions. The numerous traditional treatment options for acne, such as topical, systemic, and physical treatments, are well-known; nonetheless, drug/antibiotic-associated resistance remains challenging. In addition, monotherapy techniques are not promising for the comprehensive treatment of acne. Besides, dermatologists and other medical professionals continue to pursue successful novel drug candidates, innovative treatment methods, or combination therapies that could help better manage acne.

## Research involving human participants and/or animals

Not applicable.

## Informed consent

Not applicable.

## Declaration of competing interest

The authors declare that they have no known competing financial interests or personal relationships that could have appeared to influence the work reported in this paper.

Acknowlegement

Dr Raghvendra A Bohara wants to acknowledge Science Foundation Ireland (SFI), co-funded under the European Regional Development Fund under Grant number 13/RC/2073_P2

## Data Availability

No data was used for the research described in the article.

## List of abbreviations

Acne Vulgaris: AV
Insulin Growth hormone: IGH
Reactive oxygen species: ROS
Benzoyl Peroxide: BPO
Nitric oxide: NO
